# Descriptive epidemiology of screen and non-screen sedentary time in adolescents: a cross sectional study

**DOI:** 10.1186/1479-5868-7-92

**Published:** 2010-12-31

**Authors:** Tim S Olds, Carol A Maher, Kate Ridley, Daniella M Kittel

**Affiliations:** 1 Health and Use of Time (HUT) Group, Sansom Institute for Health Research, University of South Australia, GPO Box 2471, Adelaide, 5001, South Australia, Australia; 2 School of Education, Flinders University, GPO Box 2100, Adelaide, 5001, South Australia, Australia; 3 School of Health Sciences, University of South Australia, GPO Box 2471, Adelaide, 5001, South Australia, Australia

## Abstract

**Background:**

Much attention has been paid to adolescents' screen time, however very few studies have examined non-screen sedentary time (NSST). This study aimed to (1) describe the magnitude and composition of screen sedentary time (SST) and NSST in Australian adolescents, (2) describe the socio-demographic correlates of SST and NSST, and (3) determine whether screen time is an adequate surrogate for total sedentary behaviour in this population.

**Methods:**

2200 9-16 year old Australians provided detailed use of time data for four days. Non-screen sedentary time (NSST) included time spent participating in activities expected to elicit <3 METs whilst seated or lying down (other than sleeping), excluding screen-based activities (television, playing videogames or using computers). Total sedentary time was the sum of screen time and NSST.

**Results:**

Adolescents spent a mean (SD) of 345 (105) minutes/day in NSST, which constituted 60% of total sedentary time. School activities contributed 42% of NSST, socialising 19%, self-care (mainly eating) 16%, and passive transport 15%. Screen time and NSST showed opposite patterns in relation to key socio-demographic characteristics, including sex, age, weight status, household income, parental education and day type. Because screen time was negatively correlated with NSST (r = -0.58), and exhibited a moderate correlation (r = 0.53) with total sedentary time, screen time was only a moderately effective surrogate for total sedentary time.

**Conclusions:**

To capture a complete picture of young people's sedentary time, studies should endeavour to measure both screen time and NSST.

## Background

There has been considerable focus recently on sedentary behavior as a risk factor for negative physical and mental health outcomes in children and adults independent of physical activity [1,2]. Commonly, screen sedentary time (SST) -- and particularly time spent watching television -- is used as a surrogate for sedentary behaviours in general [3,4]. Television is the dominant screen behaviour, constituting about 70% of all SST for children [5,6]. With the high prevalence of overweight and obesity, physical activity guidelines now commonly include SST limits, on the basis that excessive SST may contribute to the problem. A variety of mechanisms linking SST and weight status have been proposed, including the possibility that SST may displace more active pursuits [7], the observation that television time is associated with increased snacking [8], and the fact that television viewing is known to increase exposure to advertisements for high energy density foods [9-11], which has been shown to influence food choices at other times of the day [12]. Sisson and colleagues [13] recently found that nearly half (47%) of US children and adolescents exceeded the recommended two hour daily limit of SST.

From the point of view of researchers wishing to design interventions, SST is an attractive target for several reasons. Increased SST is known to be associated with excessive adiposity in children [6,14], thus reducing SST may help address the issue of childhood overweight and obesity. Screen time is relatively discrete, easily identified, and cheap to measure (in comparison to physical activity which is often measured using relatively expensive instruments such as accelerometers and doubly labeled water). These advantages make it a cost-effective and clear target for surveying, monitoring and parental regulation. Furthermore, SST is seen largely as discretionary time, a "time buffer" which exhibits considerable elasticity to competing demands, and hence is a good target for behavioural interventions.

However, SST is not the only form of sedentary behaviour in adolescents, who also spend substantial amounts of time sitting in school classes, riding in cars, eating, socialising, reading and studying [5]. This non-screen sedentary time (NSST) is relatively under-researched. The underlying assumption in behavioural epidemiology in this area is that SST is a good surrogate for sedentary behaviour in general, that either SST quantitatively dominates sedentary behaviour [15,16], or that patterns of SST (in relation to socio-demographic and health-related characteristics, for example) are similar to patterns of overall sedentary behaviour [17,18]. In adults, Sugiyama and colleagues found that television time was associated with time in other sedentary behaviours in women, but not in men [19]. Amongst UK adolescents, Biddle and colleagues [20] found that television time was negatively associated with other leisure time sedentary behaviours (comprising computer use, sitting and talking, hanging out, listening to music, reading, phone, behavioural hobbies and homework), prompting them to conclude that television viewing did not reflect additional time in other sedentary behaviours. However, it is unclear from Biddle et al.'s (2009) study whether overall SST might be an adequate surrogate for total sedentary time, particularly in light of the exclusion of sedentary activities undertaken at school, on the basis that these activities are not discretionary.

The aims of this study were to (1) describe the magnitude and composition of screen sedentary time and NSST in a random sample of Australian adolescents, (2) describe the socio-demographic, temporal and personal correlates of SST and NSST, and (3) determine whether SST is a suitable surrogate for sedentary behaviour in this population.

## Methods

Subjects were 2200 randomly selected Australians aged between 9 and 16 years, who took part in the 2007 Australian National Children's Nutrition and Physical Activity Survey (Table 1). The details of the sampling, recruitment strategy and methods of the survey have been reported elsewhere [21]. Briefly, demographic data, including reported annual household income, parental education level, sex and age of the target child were gathered during a computer-assisted face-to-face interview in subjects' homes. Height, body mass and waist girth were measured according to the protocols of the International Society for the Advancement of Kinanthropometry [22], and body mass index (BMI) was calculated.

**Table 1:** Subject characteristics. Values are shown as percentages or means (SDs).

	Boys	Girls	All
**n**	1089	1111	2200
**Age (years)**	13.5 (2.2)	13.4 (2.2)	13.4 (2.2)
**BMI (kg.m^-2^)**	20.4 (3.9)	21.1 (4.2)	20.7 (4.1)
**% obese**	5.8	7.1	6.5
**% overweight (not including obese)**	17.5	20.5	19.0
**SEIFA**	1004 (66)	1000 (63)	1002 (65)

BMI = Body Mass Index

SEIFA = Socio-economic indicators for areas. SEIFA is a series of indexes of socio-economic status devised by the Australian Bureau of Statistics using a range of indicators such as educational and employment status. The index used here is the Index of Relative Disadvantage, calculated at the postal area level. The national average is 1000 and the SD is 100. Higher values indicate more advantaged areas.

Use of time data were collected using the Multimedia Activity Recall for Children and Adolescents (MARCA) [23]. The software allowed young people to recall everything they did on the previous day from wake-up to bedtime, in time-slices as fine as 5 minutes, using a segmented day format. Young people chose from a list of about 250 activities grouped under seven rubrics (Inactivity, Transport, Sport and Play, School, Self-Care, Chores and Other). The MARCA has a same-day test-retest reliability of r = 0.84-0.92 for major outcome variables [moderate to vigorous physical activity (MVPA), physical activity level (PAL) and SST)], and criterion validity with reference to accelerometry of rho = 0.45 for PAL [23] and rho = 0.54 with reference to pedometry [24]. The MARCA was administered on two occasions. Each time, young people recalled their activities over the two previous days (i.e. a total of four days were sampled). Wherever possible, at least one school day and one non-school day were sampled.

### Data treatment

NSST was calculated as the number of minutes the adolescent reported being involved in activities when seated or lying down expected to elicit <3 METs, as listed in the MARCA compendium [25] with the exception of sleep. While some have suggested that sedentary time may be defined on the basis of energy expenditure as 1.0-1.5 METs (with 1.6-2.9 METs classified as light activity) [26], for the purpose of this paper we defined sedentariness in terms of activity type, based on the meaning of the original Latin term *sedere *('to sit') [27]. We felt it was important to include all sitting activities <3 METs since inactivity physiology research has found differences in cellular mechanisms, such as electromyogram patterns, across sitting, standing and locomotive activities [2]. However, it is noteworthy that the seated activities with an energy expenditure >1.5 and <3.0 METs, such as sitting and talking, contributed only 2.2% of total NSST minutes. Seated activities which were excluded from NSST based on energy expenditures >3 METs included playing drums (4.0 METs) or trombone (3.5), horseback riding (4.0) and cycling (4.7).

Screen time was the number of minutes the adolescent reported watching television, playing videogames or using a computer. Total sedentary time was calculated as the sum of SST and NSST, although it should be noted that this time is not strictly speaking sedentary (that is, performed while seated), since adolescents often watch television, eat, read, study and otherwise conduct their lives lying down. Some seated activities exceeded the 3 MET limit and thus were excluded. Since Australian children spend about one day in two at school, overall SST, NSST and total sedentary time were calculated as the average of school day and non-school day values.

Reported household income was stratified into four bands: >AUD104,000; AUD75,000-104,000; AUD52,000-75,000; and <AUD52,000, based on Australian Bureau of Statistics classifications. Education level was based on the highest level reported by either caregiver, and categorised as high school only, some post-secondary education (e.g. trade certificate or diploma), or university degree. Remoteness of residence was stratified into four bands using the Accessibility/Remoteness Index for Australia ("ARIA+") method [28]: major city, inner regional, outer regional and remote.

Weight status category (thin, normal weight, overweight or obese) was calculated using the criteria of Cole et al. [29].

### Statistical analysis

The magnitudes of SST, NSST and total sedentary time were described as means and standard deviations. The relationships between socio-demographic characteristics and SST, NSST and total sedentary time were determined using one-way ANOVA (for categorical variables) or regression (continuous variables). To examine whether SST was an adequate surrogate for total sedentary time, Pearson's r was used to quantify the strength of the relationship between SST and total sedentary time. Further analysis using linear regression (for continuous independent variables) and ANOVA (for categorical independent variables) tested whether the residuals were significantly associated with other socio-demographic or use of time variables. High and low SST categories, and high and low total sedentary time categories were created using median splits, and cross-tabulation was used to calculate the degree to which adolescents with high total sedentary time could be identified using SST as an index. Except for when comparisons were made across sexes and ages, values for SST, NSST and total sedentary time were adjusted for age and sex by regressing them against decimal age and fitting a fourth-order polynomial. This was done separately for boys and girls, and the residuals were used in analysis where appropriate. Paired t-tests were used to compare SST, NSST and total sedentary time on school vs. non-school days. Alpha was set at 0.05. No corrections were made for multiple comparisons, but exact p-values have been reported.

## Results

### The magnitude of non-screen, screen and total sedentary time

Adolescents spent 345 ± 105 minutes/day (mean ± SD) in NSST, 230 ± 114 minutes/day in SST, and thus 575 ± 101 minutes/day in sedentary activities in total. Overall, NSST constituted 60% of total sedentary time. The average duration of a bout of NSST was 28.5 minutes. The longest reported bouts of continuous sitting were for social talking (e.g. at parties and get-togethers, 123 minutes), part-time work (105 minutes), cinema (100 minutes), knitting and sewing (63 minutes) and watching live sporting events (62 minutes).

**Figure 1:**
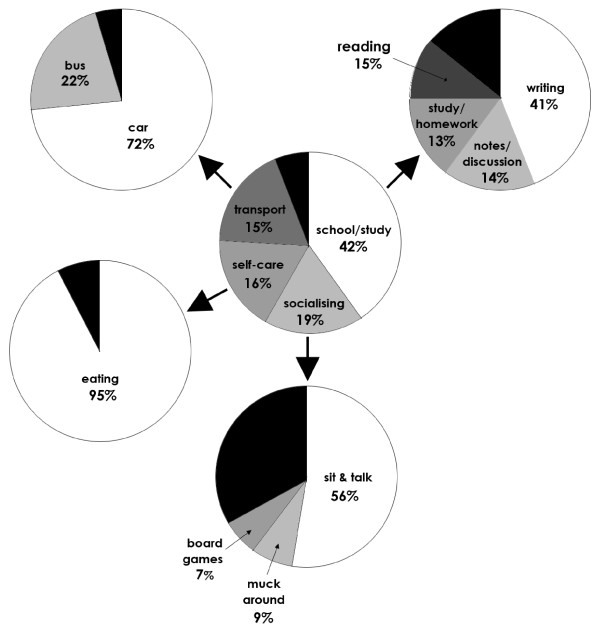
**The components of non-screen sedentary time (NSST)**. The central circle represents all NSST divided into the major domains, and the peripheral circles the contributors within each domain. The unlabeled black areas represent the total of minor contributors within each domain.

More than one-third (42%) of NSST consisted of school-based or study activities. Of this, 41% was spent writing, 15% reading, 14% taking notes or class discussion, and 13% study or homework. The next biggest contributors were social activities (19% of NSST), of which sitting and talking constituted the largest part (56%), followed by "mucking around" (non-specific seated activity; 9%) and board and card games (7%). Self-care constituted 16% of all NSST, to which the overwhelmingly greatest contributor was eating (95%). Passive transport, mainly by car (72%), constituted 15% of NSST (Figure 1).

### The relationship between non-screen, screen and total sedentary time and socio-demographic and personal variables

**Figure 2:**
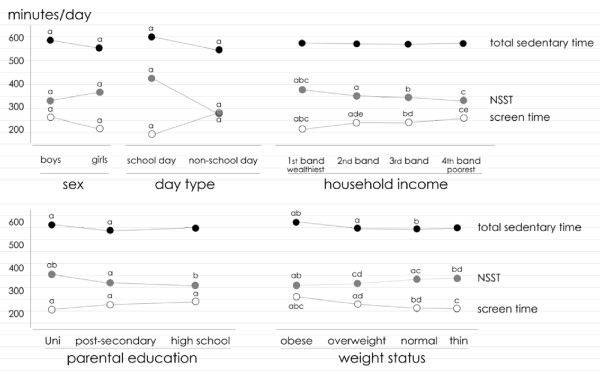
**Relationships between socio-demographic variables and total sedentary time (black dots), non-screen sedentary time (NSST; grey dots) and screen time (white dots)**. Datapoints joined by the same line with the same superscript are significantly different from each other. Uni = university.

Table 2 shows SST, NSST and total sedentary time across various socio-demographic categories and day types. These relationships are summarised visually in Figure 2. In general, SST and NSST showed opposite socio-demographic gradients.

**Table 2:** Adjusted mean (standard deviation) values (minutes/day) for non-screen sedentary time (NSST), screen time and total sedentary time (TST) across selected socio-demographic categories.

		NSST	Screen time	TST
**All adolescents**		345 (105)	230 (114)	575 (101)
**Sex**^2^	**Boys**	328 (104)	255 (117)	584 (104)
	**Girls**	361 (102)	205 (103)	566 (94)
		**p < 0.0001**	**p < 0.0001**	**p < 0.0001**
**Day type**	**School days**	425 (102)	181 (102)	606 (102)
	**Non-school days**	270 (125)	274 (151)	544 (128)
		**p < 0.0001**	**p < 0.0001**	**p < 0.0001**
**Household income**^1^	**1^st ^band (wealthiest)**	370 (101)^abc^	204 (98)^abc^	578 (97)
	**2^nd ^band**	341 (98)^a^	232 (110)^ade^	573 (99)
	**3^rd ^band**	337 (100)^b^	234 (110)^bd^	571 (105)
	**4^th ^band (poorest)**	331 (104)^c^	248 (117)^ce^	578 (98)
		**p < 0.0001**	**p < 0.0001**	**p = 0.52**
**Parents' education**^1^	**University**	369 (104)^ab^	215 (105)^a^	584 (102)^a^
	**Some post-secondary**	332 (96)^a^	236 (111)^a^	567 (103)^a^
	**High school only**	323 (105)^b^	251 (114)^a^	573 (93)
		**p < 0.0001**	**p < 0.0001**	**p = 0.0015**
**Remoteness**^1^	**Major city**	351 (97)^ab^	234 (103)	586 (95)^abc^
	**Inner regional**	335 (104)^a^	229 (114)	564 (98)^ad^
	**Outer regional**	344 (105)^c^	220 (104)	565 (101)^be^
	**Remote**	321 (95)^bc^	219 (115)	540 (104)^cde^
		**p = 0.0015**	**p = 0.12**	**p < 0.0001**
**Weight status**^1^	**Obese**	324 (105)^ab^	274 (124)^abc^	598 (110)^ab^
	**Overweight**	335 (95)^cd^	240 (100)^ad^	574 (100)^a^
	**Normal weight**	349 (104)^ac^	224 (110)^bd^	573 (97)^b^
	**Thin**	357 (110)^bd^	222 (111)^c^	579 (101)
		**p = 0.003**	**p < 0.0001**	**p = 0.03**

Values in the same section and row with the same superscript are significantly different (p < 0.05). For NSST across household income bands, for example, the wealthiest band is significantly different form all other bands.

^1^Adjusted for age and sex

^2^Adjusted for age

### Age and sex

There were significant age-related differences in NSST (p < 0.0001). NSST decreased until the peri-pubertal years (12-14) and then increased again among older adolescents (Figure 2). The inverse pattern was seen for SST, which peaked in the peri-pubertal years. Total sedentary time rose linearly across the age bands at the rate of about 9 minutes/day per year of age, from 541 (93) minutes/day at age 9 to 603 (114) minutes/day at 16.

Boys accrued 33 fewer minutes/day of NSST than girls (p < 0.0001) when adjusted for age, however they accumulated considerably more SST (50 minutes/day more; p < 0.0001), and hence more total sedentary time (by 18 minutes/day; p < 0.0001).

### Day type

NSST was 155 minutes greater on school days than on non-school days (425 ± 102 minutes/day versus 270 ± 125 minutes/day; p < 0.0001), whereas SST was 93 minutes lower on school days (p < 0.0001). Consequently, total sedentary time was 62 minutes/day greater on school days than on non-school days (p < 0.0001).

### Household income and parental education

When adjusted for age and sex, adolescents from the highest income households experienced the highest levels of NSST, accruing 29-39 minutes/day more than adolescents from other income bands (p < 0.0001). In contrast, SST was highest in the poorest households (p < 0.0001). Total sedentary time was not significantly different across income bands (p = 0.52), varying by no more than 7 minutes/day across income bands. Similar patterns were seen for parental education. Adolescents with at least one university-educated parent accumulated 37-46 minutes day more NSST than adolescents with parents of lower educational levels (p < 0.0001). Conversely, SST was highest among children of high-school educated parents (251 minutes/day), and lowest among children of university-educated parents (215 minutes/day; p < 0.0001). There was a weak but significant (p = 0.0015) association between education level and total sedentary time, although the differences amounted to no more than 17 minutes/day.

### Geographical remoteness

NSST was significantly associated with geographical remoteness (p = 0.0015). Adolescents living in major cities accrued more NSST (351 minutes/day) than adolescents living in inner regional (335 minutes/day) or remote (321 minutes/day) areas. Screen time did not differ significantly across geographical areas, however total sedentary time did (adolescents in major cities experienced 46 minutes/day more than remote adolescents; p < 0.0001).

### Weight status

NSST was associated with weight status (p = 0.003), with leaner adolescents generally accumulating more NSST. Normal weight adolescents experienced 25 minutes/day more NSST than obese adolescents. BMI z-score was significantly (p = 0.005), but weakly and negatively (r = -0.06), associated with NSST, as was age- and sex-adjusted waist: height ratio (r = -0.05, p = 0.02).

Screen time showed the opposite gradients, being higher in obese adolescents (274 minutes/day) than in overweight (240 minutes/day) and normal weight adolescents (224 minutes/day; p < 0.0001). Total sedentary time also increased as weight status increased, from 573 minutes/day for normal weight adolescents to 598 minutes/day for obese adolescents (p = 0.03).

### Screen time vs. television time

Television time constituted about 70% of SST, and was strongly correlated with SST (r = 0.73, p < 0.0001). However, the correlations were less strong for boys (r = 0.66) than for girls (r = 0.83), and for older adolescents (r = 0.67-0.70 for 14-16 year olds, r = 0.79-0.84 for 9-13 year olds). These differences reflect differences in videogame time in boys, and computer time in older adolescents. The socio-demographic patterns which characterised SST also characterised television time. Boys experienced 10 minutes/day more television time than girls (p = 0.005), and in both sexes television time peaked at ages 12-13 years. Television time was 65 minutes greater on non-school days than on school days (p < 0.0001). Television time was inversely related to educational status and income (both p < 0.0001; adolescents from households in the wealthiest quartile accumulated 30 minutes/day less television time than adolescents from the poorest quartile). Television time increased with weight status (p < 0.0001), with obese adolescents experiencing 37 minutes more television time each day than children of normal weight.

### Is screen time an adequate surrogate for total sedentary time?

In spite of a significant negative correlation (r = -0.58, p < 0.0001) between SST and NSST, there was a moderate linear relationship between SST and total sedentary time (r = 0.53, p < 0.0001), which was relatively consistent across genders (r = 0.57 for boys, r = 0.47 for girls), but decreased slightly across age groups (from r = 0.65 for 9 year olds to r = 0.44 for 16 year olds). The residuals of the SST -total sedentary time regression were not significantly related to sex, BMI z-score or weight status category, but were related to age (p < 0.0001; older adolescents showed larger residuals); area-level SEIFA (p = 0.008; higher SES areas showed larger residuals); household income (p < 0.0001; wealthiest quartile had larger residuals); and parental education (p < 0.0001; higher parental education associated with larger residuals). Therefore, the usefulness of SST as a surrogate for total sedentary time was reduced in older adolescents of higher socio-economic status. Identifying adolescents as being in the high or low total sedentary time category (using a median split) based on their having high or low SST correctly classified them in 66% of cases (69% for boys, 64% for girls). Classificatory success decreased across age groups from 70% for 9 year olds to 61% for 16 year olds, and across age groups (79-85% across each age from 9 to 16 years). Therefore, SST proved to be only a moderately effective predictor of total sedentary time.

## Discussion

Screen time and NSST showed opposing patterns in relation to key socio-demographic factors, such as sex, age, weight status, household income, parental education and day type.

The contrasting patterns of NSST tended to compensate (at least partially) for SST across socio-demographic groups. One interpretation of this compensatory effect may be that adolescents have a homeostatic mechanism regulating sitting time, a "sedostat" analogous to the "activitystat" hypothesis proposed by Wilkin et al. [30]. Wilkin and colleagues [30] have suggested that increasing physical activity in one domain (e.g. school) may result in decreasing physical activity in another (at home). Similarly, it may be that reduction of SST will result in increased NSST. This hypothesis has synergies with the 'compensation effect' described by Biddle et al. [5] where adolescents switch between sedentary choices rather than accruing additional total sedentary time. Another possible explanation relates to behavioural economics theory, which suggests adolescents often make choices between being physically active or sedentary and are influenced by both reinforcing and constraining environmental and social factors. If such a homeostatic mechanism exists, it is unclear whether it has a physiological, environmental or social basis, or a combination of such influences [31].

Regardless, SST and NSST should not be seen as qualitatively equivalent or interchangeable. The energetic demand of SST (mean = 1.3 METs) is estimated to be somewhat lower than NSST (mean = 1.5 METs). Additionally, NSST is often considered to be more socially "valuable" than SST. From a use of time point of view, SST is much more "permeable" than NSST, that is, it allows for fragmentation (interspersing bouts of SST with other activities) and time compression (reducing total time commitment by multi-tasking). Furthermore, television time is associated with exposure to food advertising and with increased snacking, and hence perhaps with unfavourable dietary habits [8]. In contrast, time spent in sedentary homework activities has been associated with more favourable dietary habits, such as increased fruit and vegetable intake [32]. Because of these numerous differences, reducing NSST is unlikely to be functionally equivalent to reducing SST in terms of modifying energy balance.

An important question is the extent to which SST can be used as a surrogate for, or a predictor of, total sedentary time, given that SST is the most commonly measured aspect of total sedentary time. The correlation between SST and total sedentary time was moderate (r = 0.53) but highly significant. However, the standard error of estimate (86 minutes/day) was large, suggesting that in 95% of cases total sedentary time could only be predicted from SST to within about 172 minutes/day, or about 30% of mean total sedentary time. Using median splits, SST could only be used to correctly categorise adolescents into high or low total sedentary time in about two thirds of cases. The predictive power of SST varied with age, parental education, household income, and area-level SES, suggesting that it may be less useful in predicting total sedentary time in particular groups, particularly older adolescents from high SES families.

The specific strengths of this study include the large sample size, the random nature of the selection process, the wide age range, and the fact that activity was assessed using a methodology which yielded very high-resolution use-of-time data. This is also the first study to comprehensively describe NSST, and to compare it to SST. The study nonetheless has limitations. The use of time approach did not capture multi-tasking, asking adolescents to nominate the activity they were most focused on, or dividing stretches of time between two activities. The survey was conducted mainly in autumn and winter, and seasonal patterns may affect both SST and NSST. There are also well known limitations to self-report, particularly with younger children, although the instrument used has been shown to have high reliability and good validity. In this study, however, the self-reported duration of daily TST (575 minutes/day) was somewhat larger than objectively measured TST reported in young people of similar ages; i.e. ~364-450 minutes per day [33-35]. This may be because accelerometers are typically not worn throughout the entire waking day (about 15 hours in this sample). Accelerometer data are often included in analyses if the participant provides >8 h of valid recording per day. Accelerometers may be removed prior to bed and not capture time spent lying awake in bed and other sedentary aspects of the pre-bedtime routine. The choice of accelerometer cut-point used to define sedentary behaviour is also likely to influence the measurement of TST.

## Conclusions

There has been great concern over secular increases in fatness in children and adolescents. Many interventions are predicated on increasing physical activity and/or reducing sedentary behaviour as a means of improving energy balance [36,37]. Some studies have been successful in improving weight status by focusing on reducing SST [38]. Despite the inverse relationship between weight status and NSST identified in this study, scope remains for interventions targeting NSST, such as active lesson breaks in the classroom [39], or active transport to school [40].

While television time appeared to be a passable surrogate for overall SST, SST was only a moderately effective predictor of total sedentary time. Furthermore, the relationships between SST, television time and total sedentary time vary within certain subsets of the adolescent population or on certain day types. Therefore, future surveys should, as much as possible, attempt to capture NSST as well as SST for a more complete picture of sedentary behaviours in adolescents.

## Competing interests

The authors declare that they have no competing interests.

## Authors' contributions

This study involved secondary analysis of an existing large dataset. All authors participated in the conception of the study and drafting the manuscript. TO and DK carried out the statistical analysis. TO oversaw collection of the original dataset. CM coordinated drafting of the manuscript. All authors read and approved the final manuscript.
